# Comparative analysis of the daily liver transcriptomes in wild nocturnal bats

**DOI:** 10.1186/s12864-022-08823-y

**Published:** 2022-08-10

**Authors:** Yujia Chu, Jingjing Li, Lei Feng, Guoting Zhang, Hui Wu, Tinglei Jiang, Hui Wang, Jiang Feng

**Affiliations:** 1 College of Life Science, Jilin Agricultural University, Changchun, 130118 China; 2 Jilin Provincial Key Laboratory of Animal Resource Conservation and Utilization, Northeast Normal University, Changchun, 130117 China

**Keywords:** Bat, Circadian rhythm, Liver, Comparative transcriptome

## Abstract

**Background:**

Mammals rely on the circadian clock network to regulate daily systemic metabolism and physiological activities. The liver is an important peripheral organ in mammals, and it has a unique circadian rhythm regulation process. As the only mammals that can fly, bats have attracted much research attention due to their nocturnal habits and life histories. However, few research reports exist concerning the circadian rhythms of bat liver gene expression and the relevant biological clock regulation mechanisms in the liver.

**Results:**

In this study, the expression levels of liver genes of Asian particolored bats were comparatively analyzed using RNA-seq at four different time points across 24 h. A total of 996 genes were found to be rhythmic, accounting for 65% of the total number of expressed genes. The critical circadian rhythm genes *Bmal1*, *Rev-erbα*, *Cry,* and *Ror* in the liver exhibited different expression patterns throughout the day, and participated in physiological processes with rhythmic changes, including Th17 cell differentiation (ko04659), antigen processing and presentation (ko04612), the estrogen signaling pathway (ko04915), and insulin resistance (ko04931). In addition, previous studies have found that the peroxisome proliferator-activated receptor (PPAR) metabolic signaling pathway (ko03320) may play a vital role in the rhythmic regulation of the metabolic network.

**Conclusions:**

This study is the first to demonstrate diurnal changes in bat liver gene expression and related physiological processes. The results have thus further enriched our understanding of bats’ biological clocks.

**Supplementary Information:**

The online version contains supplementary material available at 10.1186/s12864-022-08823-y.

## Introduction

Circadian rhythms maintain and coordinate the internal control of biological systems, allowing the body to better adapt to periodic changes in the external environment. This process has been refined by natural selection in the long-term evolutionary process [1, 2]. Studies have shown that animal circadian rhythms are primarily driven and regulated by master circadian clock genes in the suprachiasmatic nucleus (SCN) of the hypothalamus [3]. For example, the expression of Per1 increases with increasing light intensity, and causes a phase shift of SCN neural molecules [4]. In addition, the peripheral clock, composed of the liver, kidney, and other peripheral organs, also plays an important role in controlling and maintaining the daily rhythms of the animal body [5–7].

Among the peripheral organs, the liver is important for metabolic regulation and detoxification in mammals. The oscillating rhythms of liver genes are unaffected by other organs under certain conditions [8, 9]. The circadian clock of the liver, as a genomic program for the expression of liver genes, controls the rhythmic expression of many physiological and biochemical processes such as the sleep–wake cycle, the fasting-eating cycle, glucose homeostasis [10, 11], detoxification processes [12], lipid [13] and bile acid metabolism [14], and ribosome assembly and protein synthesis [15, 16]. Relevant studies have shown that the biological clock of the liver may have different regulatory modes in different organisms [17]. At present, research on the biological clock of the liver has focused on model animals, such as mice, and there are few studies on wild nocturnal mammals. As the only wild nocturnal mammals that can fly, bats occupy a vast niche in the night sky and have evolved a unique echolocation ability [18, 19]. Recent years, have seen rich and in-depth research on echolocation behavior and disease physiology of bats. The molecular basis and evolutionary mechanisms of bats’ unique nocturnal habits and behavior patterns remain to be studied.

To reveal the molecular mechanism that regulates the circadian rhythms of bats, we employed Asian particoloured bats (*Vespertilio sinensis*) and used RNA-seq to detect and compare the expression levels of liver genes at different time points during the day. We analyzed the differential expression of genes in different activity states, focusing on 1) the composition and differences of expressed genes in bat liver tissues at different time points, 2) the periodic oscillation rhythm of differentially expressed genes in Asian particoloured bats liver tissues within 24 h, 3) rhythm changes in important physiological and biochemical processes involving differentially expressed genes at different time nodes, and 4) screening and identifying important circadian clock regulatory genes in the liver of Asian particolored bats. The results of this study are expected to reveal the transcriptional regulation mechanism of biorhythmic genes in bat liver tissues and to provide new insights into the origin and evolutionary dynamics of nocturnal mammals.

## Results

### Sequence analysis, assembly, and functional annotation

We obtained the raw data by sequencing and obtained clean reads after removing low-quality sequences. The analysis for periods of satiation, sleep, fasting, and activity yielded 43,367,456, 41,534,046, 43,385,119, and 46,479,130 clean reads, respectively (Table 1). The proportion of clean reads in the original data was between 95.39% and 96.36%, indicating the high quality of the sequencing. The liver and brain clean reads were assembled together, and a total of 403,707 unigenes were obtained that were used as a reference for gene expression analysis. The results of correlation analysis between samples (Figure S1) and principal component analysis (Figure S2) indicated that the samples taken at the same time points had good repeatability and could be used for subsequent gene differential expression analysis.

**Table 1:** Sequencing, assembly, and mapping statistics of liver samples for four states

	Satiation	Sleep	Fasting	Activity
Sequencing				
Total Sequences(bp)	5,912,787,007	5,645,270,383	5,866,972,382	6,308,494,389
Total reads(raw reads)	45,188,738	43,106,938	45,486,392	48,565,592
Clean reads	43,367,456	41,534,046	43,385,119	46,479,130
Ratio of clean/raw	95.97%	96.36%	95.39%	95.71%
Assembly				
Unigenes	276,121			
N50	615			
Max. length	19,402			
Ave. length	511			
Mapping				
Total mapped reads(%)	64.95%	64.16%	65.41%	75.30%
Unique mapped reads(%)	60.06%	59.46%	60.13%	65.37%
Unigenes	111,380	110,265	111,502	121,224

### Differential expression analysis

**Fig. 1:**
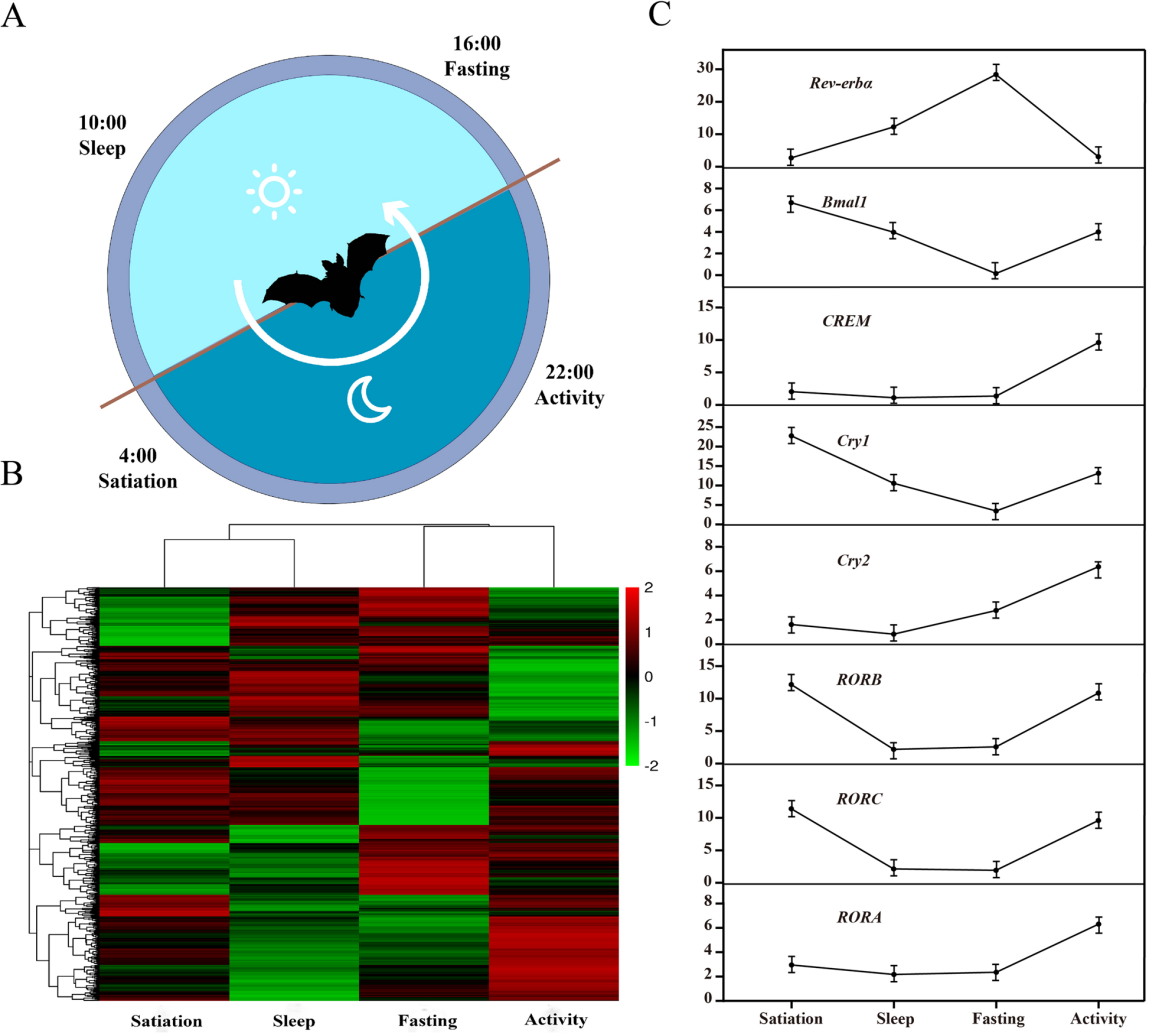
The activity of bats and the differential expression of genes in the four states. **A**. Twenty-four-hour activity pattern of Vespertilio sinensis. **B**. Heatmaps of DEGs for four states. **C**. The trend of core clock genes for four states

All expressed genes obtained at the four time points were compared, and a total of six comparison groups of differentially expressed genes were obtained. Among the six comparison groups, the number of DEGs for satiation vs. fasting involved the most genes at 628. The second highest comparison was sleep vs. activity with 524 DEGs, while the other four comparison groups had relatively fewer DEGs (Table 2). A total of 1,537 significant DEGs were obtained in the six different comparison groups; these are illustrated in the form of a heat map (Fig. 1B).

**Table 2:** The number of upregulated and downregulated differentially expressed genes in the six comparison groups, and the number of GO categories and KEGG pathways that are significantly enriched in differentially expressed genes (adjust *p*-value < 0.05)

	differentially expressed genes	GO term			KEGG pathway
	(DEGs)	Cellular component	Molecular function	Biological process	Up/Down
	Up/Down	Up/Down	Up/Down	Up/Down	
Satiation vs Sleep	135/134	23/19	25/61	58/103	6/1
Sleep vs Fasting	207/212	28/5	40/31	86/183	7/5
Fasting vs Activity	163/218	35/30	44/36	125/180	2/6
Activity vs Satiation	75/70	35/14	31/19	64/66	6/8
Satiation vs Fasting	318/310	40/22	45/80	161/362	6/4
Sleep vs Activity	223/301	21/48	43/56	94/248	2/9

The results of functional enrichment analysis of DEGs in the six comparison groups were similar to the results for the number of DEGs. The numbers of pathways that were significantly enriched in DEGs in the satiation vs. fasting and sleep vs. activity comparison groups were greater than those in the other comparison groups (Table 2).

**Fig. 2:**
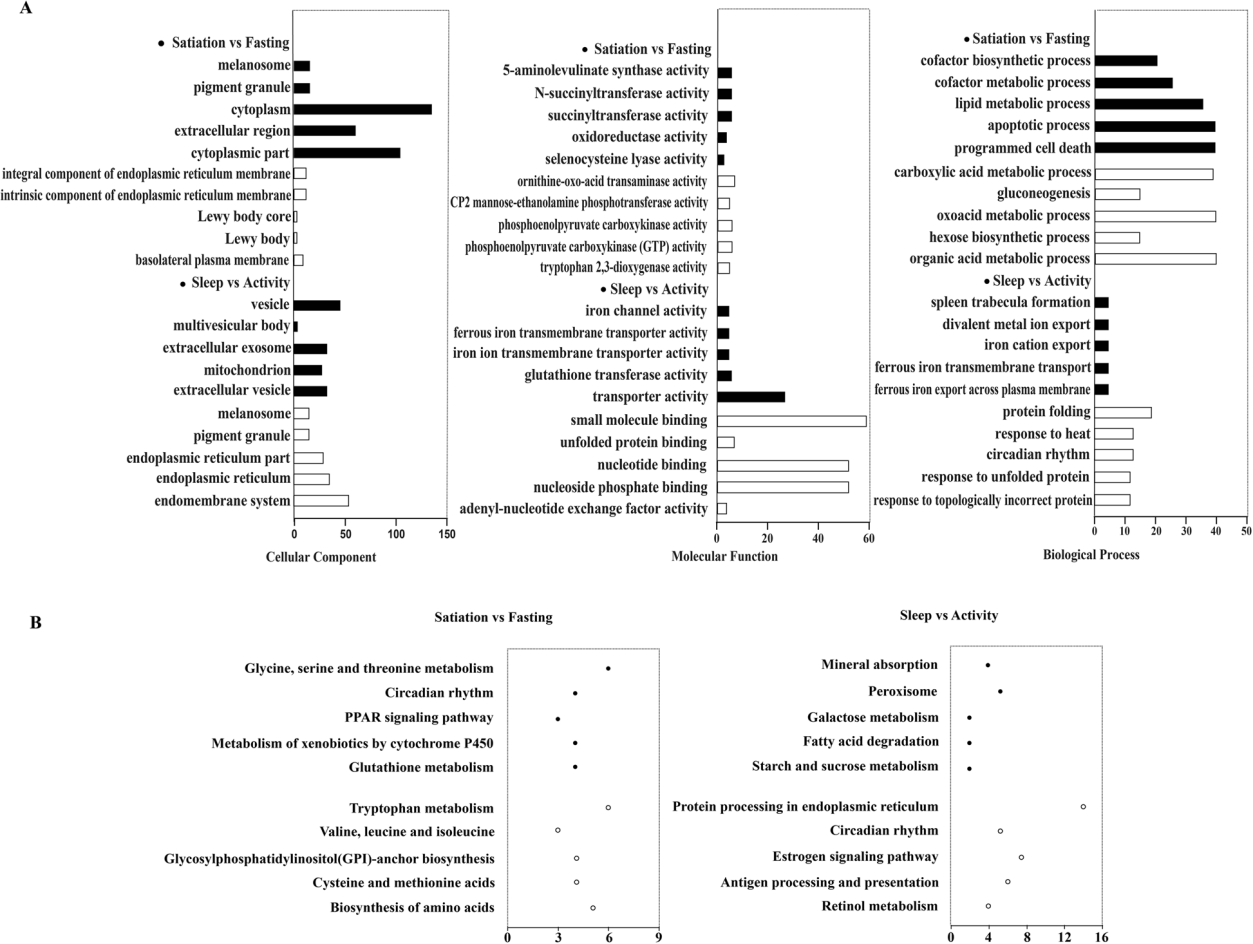
Functional enrichment analysis results of the satiation vs. fasting and sleep vs. activity groups. **A**. The top five GO categories with the most significant *p*-value were significantly enriched for upregulated genes in satiation vs. fasting and sleep vs. activity groups. **B**. The top five KEGG pathway analysis of significantly enriched genes in satiation vs. fasting and sleep vs. activity groups. The black part represents items with significant enrichment of upregulated genes. The blank part represents items with significant enrichment of downregulated genes

In the satiation vs fasting comparison group, genes overexpressed in the satiation state were significantly enriched in items related to melanosomes, pigment granules, cofactor biosynthetic processes, cofactor metabolic processes, lipid metabolic processes, and N-succinyltransferase activity. These genes were also significantly enriched in glycine, serine, and threonine metabolism (ko00260), circadian rhythm (ko04710), PPAR signaling pathway (ko03320), metabolism of xenobiotics by cytochrome P450 (ko00980), and glutathione metabolism (ko00480). Genes highly expressed in the fasting state were significantly enriched in carboxylic acid metabolic processes, gluconeogenesis, oxoacid metabolic processes, integral components of endoplasmic reticulum membrane, and ornithine-oxo-acid transaminase activity, among other processes. These genes were also significantly enriched in amino acid synthesis pathways related to metabolism such as tryptophan metabolism (ko00380), valine, leucine and isoleucine biosynthesis (ko00290), glycosylphosphatidylinositol (GPI)-anchor biosynthesis (ko00563), and cysteine and methionine metabolism (ko00270). For additional details, see the legends for Fig. 2 and Tables S1, S2, S3, and S4. The important circadian rhythm genes *Bmal1*, *Rev-erbα*, *Ror*, *Cry1*, and *Cry2* were differentially expressed in these two states, with *Rev-erbα* expression being inhibited. All listed genes participate in regulating circadian rhythm (ko04710).

In the sleep vs. activity comparison group, genes overexpressed in the sleep state were significantly enriched in related items such as spleen trabecula formation, iron channel activity, vesicle and extracellular exosomes, mineral absorption (ko04978), peroxisomes (ko04146), and other related channels. Genes overexpressed in the activity state were significantly enriched in protein folding, response to heat, melanosomes, pigment granules, endoplasmic reticulum, small molecule binding, unfolded protein binding, and nuclear binding among other terms. These genes were also significantly enriched in protein processing in the endoplasmic reticulum (ko04141), circadian rhythm (ko04710), estrogen signaling pathway (ko04915), antigen processing and presentation (ko04612), retinol metabolism (ko00830), protein export (ko03060), B cell receptor signaling pathway (ko04662), hematopoietic cell lineage (ko04640), fatty acid biosynthesis (ko00061), and other related pathways. For additional details, see the legends for Fig. 2 and Tables S1, S2, S3, and S4. The expression levels of *CREB*, *Ror*, *Cry1*, and *Cry2* were inhibited, and *Rev-erbα* were significantly increased. The above listed genes were all enriched in circadian rhythm (ko04710).

The functional enrichment results of DEGs in the four comparison groups of satiation vs. sleep, sleep vs. fasting, fasting vs. activity, and activity vs. satiation time points showed that overexpressed DEGs were significantly enriched in folding, sorting, and degradation, the endocrine system, the immune system, energy metabolism, lipid metabolism, and the digestive system. Additional related pathways were enriched, including protein processing in the endoplasmic reticulum (ko04141), thyroid hormone synthesis (ko04918), NOD-like receptor signaling pathway (ko04621), oxidative phosphorylation (ko00190), biosynthesis of unsaturated fatty acids (ko01040), and mineral absorption (ko04978). For additional details, see Table S4. For the DEGs enriched in circadian rhythm (ko04710) in satiation vs. sleep, i.e., in the satiation state, the expression of *Rev-erbα* was inhibited, while the expression levels of *Cry* and *Ror* were increased. The expression of the above genes in the fasting vs. activity comparison showed the opposite pattern.

### Short time-series expression miner analysis of DEGs

**Fig. 3:**
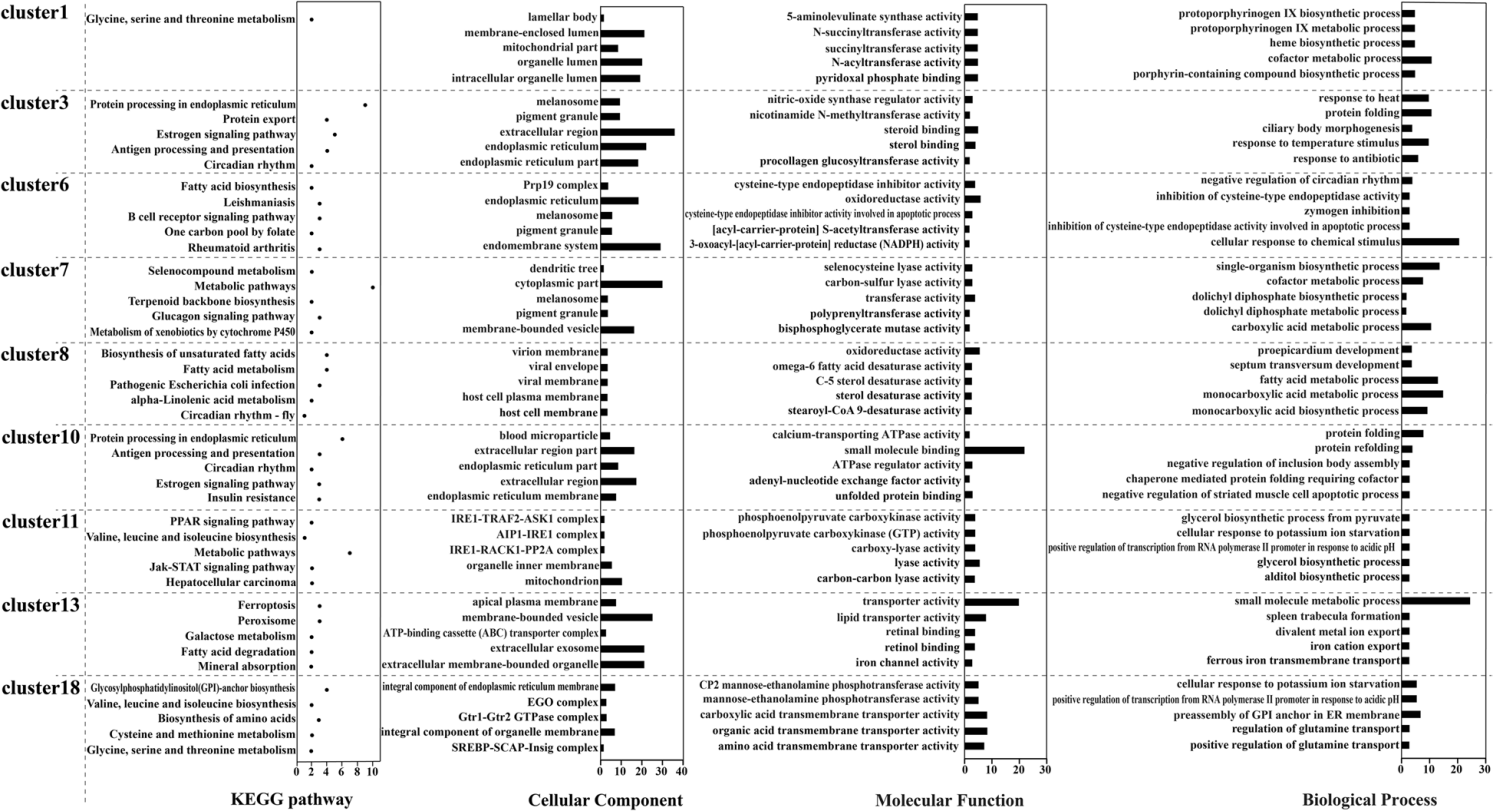
Functional enrichment analysis results of genes in nine significant clusters. The four parts from left to right represent the top five GO terms and KEGG pathway with the most significant *p*-value being significantly enriched for genes in each cluster

The short time-series expression miner (STEM) analysis of genes with similar expression patterns showed that 1,537 DEGs were divided into 20 clusters, of which nine clusters named as cluster 1, cluster 3, cluster 6, cluster 7, cluster 8, cluster 10, cluster 11, cluster 13, and cluster 18 were clusters with a significant *p-*value and were considered to represent a group of genes whose expression trends were similar throughout the day. Among these groups, cluster 1–18, cluster 6–13, and cluster 8–11 showed completely opposite trends (additional details are given in Figure S3). The results of functional enrichment analysis of the genes contained in the above nine clusters are shown in Table 3 and Fig. 3, and additional details are available in Tables S5, S6, S7, and S8.

**Table 3:** The number of GO categories and KEGG pathways significantly enriched by genes from significantly enriched genes clusters (adjust *p*-value < 0.05)

	GO term			KEGG pathway
	Cellular component	Molecular function	Biological process	
cluster1	4	20	102	2
cluster3	61	86	413	23
cluster6	36	84	265	31
cluster7	30	57	54	12
cluster8	51	53	264	19
cluster10	21	54	94	12
cluster11	6	22	165	7
cluster13	30	93	106	8
cluster18	28	104	208	9

The genes in cluster 6 began to decline in expression from satiation to sleep and gradually increased from sleep to activity. The genes in cluster 6 were significantly enriched in the entries related to endoplasmic reticulum, melanosomes, negative regulation of circadian rhythm, inhibition of cysteine-type endopeptidase activity, and oxidoreductase activity. Additional significantly enriched terms included fatty acid biosynthesis, (ko00061), rheumatoid arthritis (ko05323), B cell receptor signaling pathway (ko04622), transcriptional dysregulation in cancers (ko05202), insulin signaling pathway (ko04910), and the IL-17 signaling pathway (ko04657). The expression of genes that inhibit apoptosis and negative regulation of circadian rhythm decreases and then gradually rises as the organism begins to move. The change trend of cluster 13 was the complete opposite to that of cluster 6. The genes were significantly enriched in related items such as apical plasma membrane, ATP-binding cassette (ABC) transporter complex, small molecule metabolic process, spleen trabecula formation, transporter activity, and retinal binding, among others. Additional terms included ferroptosis (ko04216), peroxisome (ko04146), fatty acid degradation (ko00071), mineral absorption (ko04978), p53 signaling pathway (ko04115), glycine, serine and threonine metabolism (ko00260), glycolysis/gluconeogenesis (ko00010), and others. As the organism enters the sleep period the expression of genes in the above pathways increases, and as the organism's activity level increases the expression of these genes decreases.

The expression levels of genes in cluster 8 remained unchanged from satiation to sleep, then decreased from sleep to fasting and increased from fasting to activity. The genes in cluster 8 were significantly enriched in virion membrane, proepicardium development, fatty acid metabolic process, oxidoreductase activity, and C-5 sterol desaturase activity. Other related terms that were significantly enriched included biosynthesis of unsaturated fatty acids (ko01040), African trypanosomiasis (ko05143), salivary secretion (ko04970), PPAR signaling pathway (ko03320), calcium signaling pathway (ko04020), vascular smooth muscle contraction (ko04270), neuroactive ligand-receptor interaction (ko04080), and circadian rhythm-fly (ko04711) among others. From satiation to sleep, the expression of genes in the above pathways remained stable and showed a trend of first declining and then rising as the organism entered fasting and activity phases.

The genes in cluster 3 were significantly enriched in related items such as melanosomes, pigment granules, response to heat, ciliary body morphogenesis, and steroid binding. Other significantly enriched terms included protein processing in endoplasmic reticulum (ko04141), PPAR signaling pathway (ko03320), antigen processing and presentation (ko04612), the IL-17 signaling pathway (ko04657), PI3K-Akt signaling pathway (ko04151), circadian rhythm (ko04710), and other pathways. The expression levels of genes in the above pathways declined from satiation to sleep, remained stable from sleep to fasting, and began to rise after entering activity. The change trend was consistent with the activity law of the organism in response to external stimuli.

The genes in cluster 10 were significantly enriched in blood microparticles, extracellular region, endoplasmic reticulum, protein folding, negative regulation of inclusion body assembly, calcium-transporting ATPase activity involved in regulation of cardiac muscle cell membrane potential, small molecule binding, and ATPase regulator activity, and other related items. Additional significantly enriched terms included protein processing in endoplasmic reticulum (ko04141), antigen processing and presentation (ko04612), circadian rhythm (ko04710), estrogen signaling pathway (ko04915), insulin resistance (ko04931), prion diseases (ko05020), influenza A (ko05164), and other pathways. In the above pathways, gene expression was relatively stable from fullness to fasting, then began to rise during activity where the genes were highly expressed to maintain the normal activities of the body. A number of genes were enriched in pathways related to immune diseases.

The functional enrichment analysis showed that genes in each cluster were enriched in a large number of pathways, and a number of pathways or genes had the same enrichment in different clusters, or the same genes were enriched in different pathways. The circadian pathway was enriched in cluster 3, cluster 8, and cluster 10, and the core circadian clock gene *Bmal1* and the circadian supplementary regulation genes *Ror* and *CREB* were screened. *Ror* and *CREB* also participated in other non-circadian pathways (Fig. 1C and Table S8) and were enriched in pathways such as antigen processing and presentation (ko04612), estrogen signaling pathway (ko04915), insulin resistance (ko04931), and Th17 cell differentiation (ko04659). Further analysis of the results of functional enrichment revealed that the signal transduction pathways were enriched in each cluster, and a large number of signal pathways were enriched in the endocrine system, immune system, cell growth, and apoptosis. The above results indicate that signaling pathways play a vital role in the regulation of circadian rhythms. Overall, not all genes showed the same expression trend as circadian genes or were regulated by circadian genes, with different genes in the same pathway showing different expression rhythms.

### Quantitative real-time PCR (qPCR) validation

**Fig. 4:**
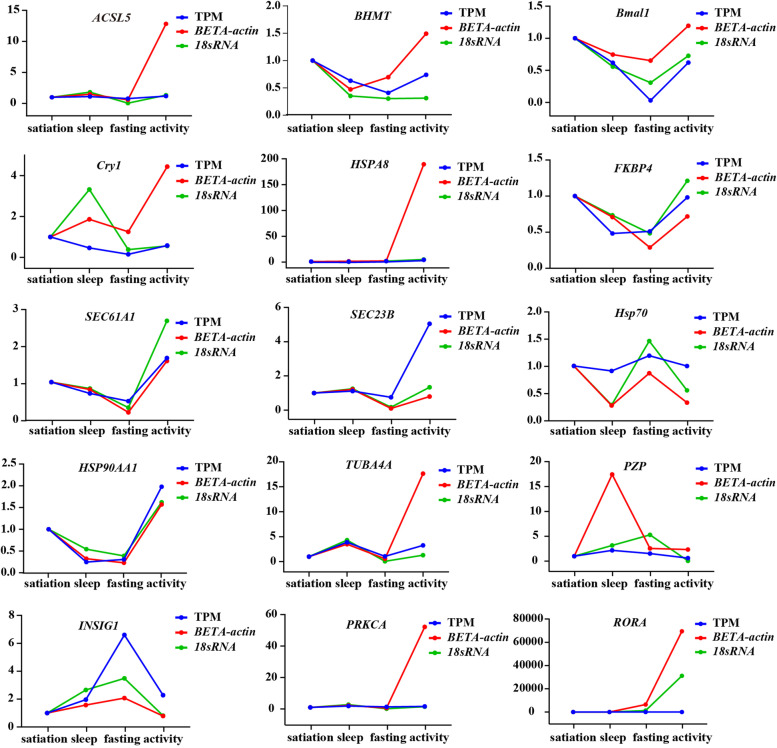
Differentially expressed genes by qPCR for technical validation

In order to confirm the reliability of the results obtained by RNA-seq, we compared the RNA-seq data for 15 randomly selected genes with the results of qPCR experiments for the changes in mRNA expression of selected genes in the four states. All tested target genes showed expression patterns similar to the results obtained by RNA-seq. In addition, the significant correlation between log_2_ fold change values detected by RNA-seq and qPCR indicated that the two independent measurements were consistent (*p* < 0.05) (Fig. 4). Our qPCR verification confirmed the reliability of the RNA-seq data.

## Discussion

By analyzing the RNA-seq data obtained from the liver of Asian particolored bats under different activity states, we found that genes expressed in the bats’ liver could show expression level changes related with biological states during the day, and the differentially expressed genes displayed a variety of oscillation patterns. Notably, some genes had autonomous rhythms and also participated in the regulation of bats’ liver functions. Several critical circadian clock related genes, including *Bmal1* and *Rev-erbα*, showed rhythmic changes during the day, and they may also participate in the regulation of various biological processes such as circadian rhythm (ko04710), alanine, aspartate and glutamate metabolism (ko00250), and insulin resistance (ko04931). Overall, number of genes and various related biological processes were revealed, indicating complex rhythmic activities in bats’ liver.

Previous studies have documented that external stimulus signals and behaviors, such as feeding and sleep could affect the expression of liver genes, especially genes related to energy storage and consumption [20–22]. When observing bats’ liver in this study, satiation vs. fasting and sleep vs. activity represented two comparisons influenced by feeding and sleep, respectively. Both groups of DEGs from satiation vs. fasting and sleep vs. activity comparisons were enriched in similar KEGG pathways related with metabolic processes, such as tryptophan metabolism (ko00380) and retinol metabolism (ko00830). In addition, the overexpressed genes detected in the fasting state compared with the satiation state were significantly enriched in other metabolism related pathways, such as valine, leucine, and isoleucine biosynthesis (ko00290), however, this metabolic process usually peaks during satiation in the liver of mice [23]. In this case, physiological precesses involved in the biosynthesis of these amino acids may exhibit different activities in the liver of mice and bats before and after feeding. Moreover, overexpressed genes detected in the activity state of bats’ liver were significantly enriched in fatty acid biosynthesis (ko00061), whereas this pathway was affected by the satiation-fasting rhythm in the liver of mice, displaying high activity in satiation and low activity during fasting [24]. In effect, the activity state in this study represented the entire active feeding process of *V. sinensis* from 6:00 p.m. until the next morning at 4:00 a.m.; therefore, we hypothesize that the activity of fatty acid biosynthesis may also be related with the satiation-fasting rhythm as in mice. However, the amount of information was limited by the sample collection time points, and thus more intensive sample collection will be needed in future studies.

Different biological processes in the bats’ liver presented various complex change patterns revealed by the results of the trend analyses. Genes grouped in cluster 3 were significantly enriched in thyroid hormone synthesis (ko04918) that displayed a lower activity level from sleep to fasting. Similar results have been observed in other nocturnal mammals, where thyroid hormone synthesis was reduced during the daytime in the liver of mice [25]. Genes grouped in cluster 7 were significantly enriched in the glucagon signaling pathway (ko04922), indicating that this pathway remained constant from satiation to sleep and then decreased. Similar results have been found in the liver of mice, where glucagon was increased after satiation and remained at a relatively high level for 4 ~ 8 h [26]. The results revealed by genes grouped in cluster 10 indicated that insulin resistance (ko04931) increased gradually from fasting to activity, and experimental studies on humans have shown that sleep–wake rhythms affect this biological process [27]. The changes in biological processes were complex; for example, genes in cluster 6 and cluster 8 exhibited different change patterns in bats’ liver during the day; however, both groups of genes were significantly enriched fatty acid metabolism (ko01212). Fatty acid metabolism exhibited a higher activity level in the activity state in both cluster 6 and cluster 8. Similar results were also observed in related studies on the liver of mice, where fatty acid metabolism was influenced by feeding behavior and usually peaked during active periods [28].

Rhythmic expression of circadian clock genes plays an important role in the regulation of daily physiology and behavior of mammals. We found that several circadian clock related genes in the liver of bats, including *Bmal1*, *Rev-erbα*, *Cry1*, *Cry2*, *Ror*, and *CREB*, were all significantly enriched in the circadian rhythm category. Moreover, these genes also presented various rhythmic expression patterns. Recently, a number of studies have shown that *Rev-erbα* is a central circadian clock gene and is a main regulator of metabolism [29–32]. On the one hand, *Rev-erbα* can regulate other circadian rhythm genes such as *Clock* and *Cry1* [33, 34]. On the other hand, it can also act as an independent transcription repressor by interacting with heme and mitochondria [35, 36]. The enrichment results of DEGs from satiation vs. sleep, satiation vs. fasting, fasting vs. activity, and sleep vs. activity indicated that *Rev-erbα* in two consecutive states, activity and satiation, presented relatively lower expression level; however, in the sleep and fasting states, *Rev-erbα* showed relatively higher expression levels. However, different situations were observed in the liver of mice, as *Rev-erbα* peaked in the satiation state, gradually decreased with time until fasting, and then gradually increased [37]. The reasons for why the rhythmic changes of *Rev-erbα* were different in bats and mice need further study. Controlled laboratory conditions for mice and the wild conditions for bats may partly account for the conflicting results. Although both bats and mice are nocturnal, different rhythmic patterns of circadian clock genes could be present in different mammal species, since there are differences in other aspects of physiology and behavior.

As one of the core circadian clock genes, *Bmal1* also had rhythmic expression in the bats’ liver (revealed by the trend analysis) and showed peak expression levels in the satiation state. The gene participates in the PI3K-AKT signaling pathway (ko04151) and calcium signaling pathway (ko04020). It has been shown that melatonin, which conveys circadian instructions in the brain, controls the expression of *Bmal1* via the PI3K-AKT signaling pathway in the brain [38]. In the bats’ livers, *Bmal1* was grouped in cluster 8 and was significantly enriched in the endocrine system, immune system, and lipid metabolism. Similar results were also found in previous studies; the expression level of *Bmal1* is regulated by receiving external signals and instructions transmitted by the SCN, which in turn affects the rhythmic biological processes described above and other related pathways as well as the expression of downstream functional genes. We also identified three other biological clock genes, *Cry1, Cry2, CREB* and *Ror,* that may be involved in the physiological circadian rhythms of the bat liver. The specific mechanisms of action of these genes should be characterized in future studies.

Generally, rats and mice are used as traditional mammalian animal models in the field of biological rhythms and biomedical research. Similar with the daily active patterns of bats, rats and mice are nocturnal (active at night), whereas humans are diurnal (active during the day) [39, 40]. Among the biological processes which were identified to be rhythmic during a day in the liver of bats, however, similar and different situations were compared with nocturnal rats and mice which were under standard conditions with a 12 light: 12 dark cycle in previous studies [23–28, 37]. Taken together, those results indicated that the daily patterns of various biological and circadian clock related genes could be similar or different in different nocturnal species, and the ecology of animal activity may partly affect the patterns of rhythmic gene expression, however, not entirely. Besides, rats and mice are usually treated with standard day and night cycles in the laboratory, which could be another reason for the different results detected in the bats collected in the wild, which need further research verification.

Overall, the DEGs detected in bats’ liver were enriched in multiple metabolic related pathways with various expression patterns. The same genes may perform various functions and thus could be enriched in multiple pathways, whereas genes with different patterns could participate in the same pathway. These expressed genes and the related pathways could affect each other and jointly maintain the stability of liver function. For example, *Ror* is involved in circadian rhythm (ko04710) and also plays a role in Th17 cell differentiation (ko04659). Fatty acid metabolism (ko01212) was significantly enriched by genes grouped in both cluster 6 and cluster 8; however, different rhythmic patterns were observed for this process. Therefore, for a single gene or pathway, it is difficult to uncover the rhythmic changes in the comprehensive biological processes and functions of the liver [41–43]. More evidence from gene expression and regulation, gene network interaction, protein synthesis, and metabolism regulation will be conducted in future studies.

## Conclusions

Using comparative RNA-seq analysis of the bats’ liver among four different biological states, a large number of DEGs were detected, and further enrichment analyses showed different degrees of rhythmic variation in areas including endocrine hormones, metabolism, and the immune system. In particular, we found that the satiation-fasting and sleep-activity cycles had a greater impact on the expression of circadian rhythm genes. Several critical circadian clock genes, *Bmal1*, *Rev-erbα*, *Cry1*, *Cry2*, *CREB*, and *Ror* in the liver exhibited different expression patterns throughout the day and participated in the physiological processes that involved rhythmic changes. Different expression patterns may influence each other to maintain the steady state of biological functions in bats’ liver. *Bmal1* and *Rev-erbα* play vital roles in the rhythmic expression of genes in the liver. The above results provide a scientific basis for further research on nocturnal animals.

## Methods and Materials

### Sample collection

This study was conducted in an Asian particolored bat colony under the overpass in Acheng District, Harbin, Heilongjiang Province (45°32′55″N, 127°32′59″E), from July to August 2020. This bat group was observed and recorded for a week, and the daily activity pattern of bats in a 24-h cycle was determined (Fig. 1A). According to the observation results, the four most representative activity states of Asian particolored bats in one day were selected for research and sampling, and the corresponding sampling time points were: 4:00 (satiation), 10:00 (sleep), 16:00 (fasting), and 22:00 (activity). All sampling procedures at the four time points were completed within one day. For the states of satiation, sleep, and fasting, bats were captured by hand-draft nets, and for the active state, bats were captured by mist nets. To reduce the sampling effects of reproduction and to avoid the influence of sex differences in the results, only non-pregnant and non-lactating females were selected. Three biological replicates were included for each time point, and 12 individuals were collected in total. For captured bat individuals, we first measured the bat's body temperature, weight, forearm length, and other basic data, after which the bats were euthanized by decapitation as soon as possible. The liver tissues from each individual were collected and flash-frozen in liquid nitrogen as soon as possible followed by placement in a − 80 °C freezer until processing for total RNA isolation.

### Transcriptome sequencing and bioinformatic analysis

#### RNA extraction and RNA-Seq sequencing

Total RNA extraction from liver samples was performed in accordance with the operating instructions of a Total RNA Extractor (Trizol) extraction kit using a Qubit 2.0 [44, 45] to detect RNA concentration. Agarose gel electrophoresis was used to detect RNA integrity and to ensure that all samples were free of genomic contamination.

To ensure the reliability of gene expression between samples, RNA samples of the same quality were taken, and the RNA fragments were used as templates to construct a cDNA library. RNA samples of the same volume and concentration were used during the conversion of mRNA into cDNA. Three paired-end cDNA libraries for each time point were generated using the mRNA-Seq assay. In total, 12 cDNA libraries were prepared at an equimolar ratio for transcriptome sequencing on an Illumina HiSeq 4000 platform. The raw sequence data generated were deposited into the NCBI Sequence Read Archive database (SRA run accession numbers: SRR15616365-SRR156163776).

### Transcriptome assembly and functional annotation

The raw reads were filtered using three criteria: removing reads with adaptors, removing reads with unknown bases “N”, and removing low-quality reads containing more than 50% low-quality bases (Q-value ≤ 20). To construct a common and powerful reference transcriptome for the comparative analyses, all high-quality raw reads from 12 individual liver cDNA libraries and 12 individual brain cDNA libraries of Asian particoloured bats were used for de novo assembly by Trinity software [46] using the default parameters. Data from the 12 individual brain cDNA libraries were analyzed in another related study. The assembled contigs with a minimum length of 200 bp were used for further analyses. Then, the CD-Hit program was used to reduce sequence redundancy of the transcriptome with the default parameter settings. All of the remaining contigs are described as unigenes in the following text.

Basic annotations of unigenes included protein functional annotation, EuKaryotic Orthologous Groups (KOG) functional annotation, Gene Ontology (GO), and pathway annotations. In detail, we used NCBI Blast + [47] with an *e-*value threshold of 1e-5 on the NCBI nonredundant protein (Nr) database (http://www.ncbi.nlm.nih.gov), the Swiss-Prot protein database (http://www.expasy.ch/sprot), the KOG database (http://www.cubi.nlm.nih.gov/KOG), and the Kyoto Encyclopedia of Genes and Genomes (KEGG) database (http://www.genome.jp/KEGG). The functional classification of unigenes was performed using the R package clusterProfiler version 3.0.5.

### Differential expression analysis

We first analyzed the genes expressed during satiation, sleep, fasting, and activity. We used Bowtie2 [48] software after processing to align the clean reads to the already assembled reference sequence. The expression levels of the genes were calculated using the number of reads that were uniquely aligned with the reference gene. Unique mapped reads were quantified into counts for each unigene, and we use the transcripts per million (TPM) [49] formula method to determine the level of expression of each unigene. The raw counts for each gene were converted into TPM value using the Salmon software based on the following formula [49]:

TPM =$$\frac{{q}_{i}/{l}_{i}*{10}^{6}}{sum({q}_{1}/{l}_{1}+{q}_{2}/{l}_{2}+\dots +{q}_{n}/{l}_{n})}$$*10^6^

TPM was introduced in an attempt to facilitate comparisons across samples. TPM stands for transcript per million, and the sum of all TPM values is the same in all samples, such that a TPM value represents a relative expression level that in principle should be comparable between samples. Here, q_i_ denotes reads mapped to a transcript; l_i_ is the transcript length, and the sum (q_1_/l_1_ + q_2_/l_2_ + … + q_n_/l_n_) corresponds to the sum of mapped reads to the transcript normalized by the transcript length.

The correlation coefficient between each pair of replicates for the four time points was calculated using the R package (version 2.16.2) to evaluate the reliability of the experimental results as well as the operational stability. To determine the separation of expression patterns across samples, principal component analysis (PCA) was performed on the levels of all unigenes using the R package gmodels version 3.4.1.

To clarify the changes in the expression of bat liver genes over one day, the genes at two adjacent time points were compared and analyzed sequentially, i.e., satiation vs. sleep, sleep vs. fasting, fasting vs. activity, and activity vs. satiation. Furthermore, two groups of the exact opposite states were compared, i.e., satiation vs. fasting and sleep vs. activity. A total of six differential gene comparison groups were obtained. The screening criteria for differentially expressed genes in each comparison group were: |log_2_ fold change|≥ 1 and a false discovery rate (FDR) < 0.05; these were considered as significantly differentially expressed genes. In terms of the definitions of upregulation and downregulation of specific genes in each pairwise comparison, an upregulated gene was defined one that had a higher expression level in the former state than in the latter, and vice versa for a downregulated gene.

### GO category and KEGG pathway enrichment analyses

Downstream functional classification was achieved through the integrated localization of GO and KEGG pathway databases. All *p*-values were computed using the hypergeometric test, and multiple test correction was performed using the Benjamini–Hochberg method based on an FDR (false discovery rate) cut-off of 0.05.

### Short Time-series expression miner analysis

To determine the overall level of gene expression in the bat liver during the day, we used STEM software to analyze the gene expression trend of all differentially expressed genes generated in the six comparison groups [50]. Differentially expressed genes (DEGs) with similar expression patterns were clustered together. For each clustered profile, DEGs that belonged to each cluster had a *p-*value < 0.05. The DEGs in the significant trend module were analyzed by GO and KEGG functional enrichment analysis to determine the biological functions and processes involved.

### Validation of sequencing data by qPCR

To confirm the expression patterns observed in our RNA-seq analysis, 15 randomly selected target genes and two housekeeping genes were used. The gene names and their primer pairs are listed in Table S9. Three replicate analyses were performed for each sample using the Applied Biosystems StepOne Real-Time PCR system. Complementary DNA (cDNA) was synthesized using the same RNA samples as for RNA-seq, and 1 μg of total RNA was reverse transcribed (Trans) using reverse transcriptase. The final reaction volume of 20 μl included cDNA (1 μl), 2xPerfectStart Green qPCR SuperMix (10 μl), Forward Primer (10 μM; 0.4 μl), Reverse Primer (10 μM; 0.4 μl), and nuclease-free water (8.2 μl). Then, PCR was performed under the following conditions: 94 °C for 30-s pre-denaturation, 94 °C for 5 s, and 60 °C for 30 s for a total of 40 cycles. The amplification efficiencies of all 19 target genes and two housekeeping genes ranged from 90 to 100%. The relative expression level of each target gene was calculated by the 2^−△△Ct^ method using the housekeeping genes as the reference standard. Regression analysis was performed to compare the qPCR expression values with the RNA-seq results.

## Supplementary Information

**Additional file 1:** **Figure S1.** Correlation analysis between each pair of replicates for four states. **Figure S2.** Principal component analysis (PCA) of the transcriptome of four states. **Figure S3.** Differential gene cluster expression trend line chart.

**Additional file 2:** **Table S1.** GO cell component enrichment data of six pairwise comparisons.

**Additional file 3:** **Table S2.** GO molecular function enrichment data of six pairwise comparisons.

**Additional file 4:** **Table S3.** GO biological process enrichment data of six pairwise comparisons.

**Additional file 5:** **Table S4.** KEGG enrichment data of six pairwise comparisons.

**Additional file 6:** **Table S5.** GO cell component enrichment data of trend cluster genes.

**Additional file 7:** **Table S6.** GO molecular function enrichment data of trend cluster genes.

**Additional file 8:** **Table S7.** GO biological process enrichment data of trend cluster genes.

**Additional file 9:** **Table S8.** KEGG enrichment data of seven cluster genes conducted by trend analysis.

**Additional file 10:** **Table S9.** Summary of qPCR primer sequences.

## Data Availability

Four sets of RNA-seq data (SRR15616365-SRR156163776) generated and/or analyzed during the current study are available in the SRA repository.

## Abbreviations

SCN: Hypothalamic suprachiasmatic nucleus
DEG: Differentially expressed gene
