# Supplementary material for: Comparative analysis of the daily liver transcriptomes in wild nocturnal bats
Source: BMC Genomics. 2022 Aug 10;23:572. doi: 10.1186/s12864-022-08823-y (PMC9367025; doi:10.1186/s12864-022-08823-y)
Supplement: Supplementary file 1 — Additional file 1: Figure S1. Correlation analysis between each pair of replicates for four states. Figure S2. Principal component analysis (PCA) of the transcriptome of four states. Figure S3. Differential gene cluster expression trend line chart. [file 12864_2022_8823_MOESM1_ESM.docx]

**Comparative Analysis of the Daily Liver Transcriptomes in Wild Nocturnal Bats**

**Yujia Chu^1^, Jingjing Li^1^, Lei Feng^2^,** **Guoting Zhang^1^, Hui Wu^1^, Tinglei Jiang^2^, Hui Wang^1^* and Jiang Feng^1, 2^***

^1^ College of Life Science, Jilin Agricultural University, Changchun 130118, China

^2^ Jilin Provincial Key Laboratory of Animal Resource Conservation and Utilization, Northeast Normal University, Changchun 130117, China

*** Correspondence:**

Jiang Feng

[fengj@nenu.edu.cn](mailto:fengj@nenu.edu.cn)

Hui Wang

wangh681@nenu.edu.cn


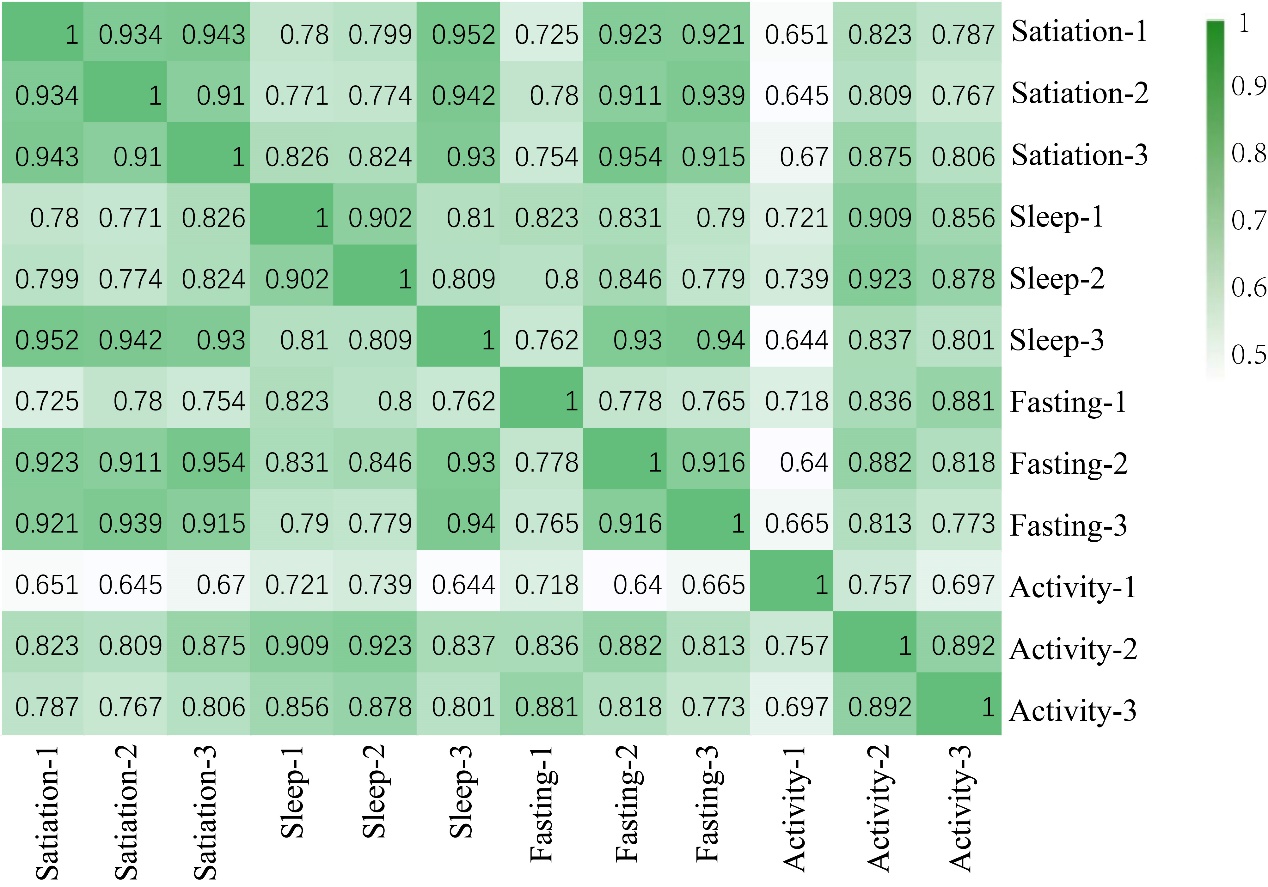


**Figure S1.** Correlation analysis between each pair of replicates for four states.


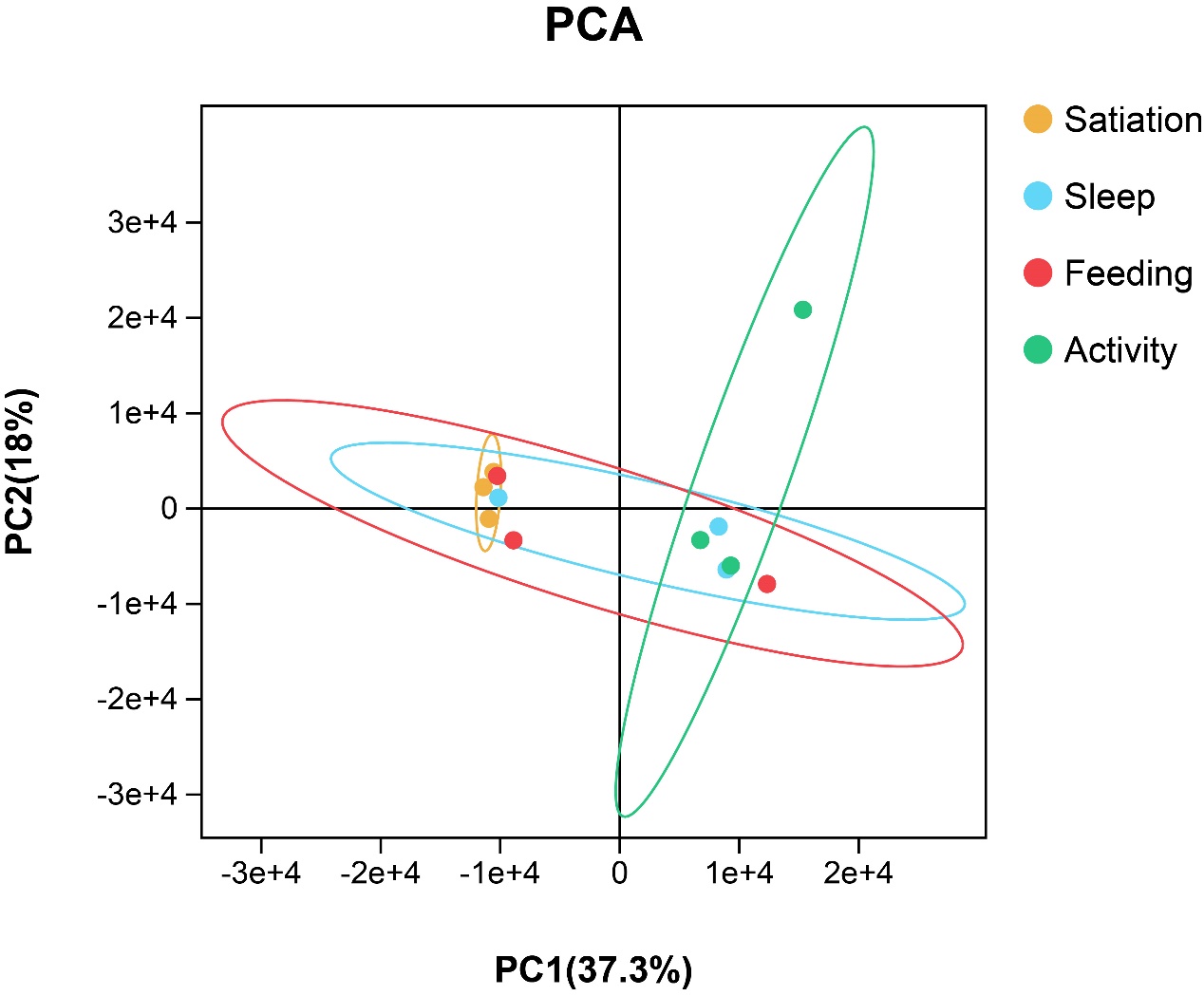


**Figure S2.** Principal component analysis (PCA) of the transcriptome of four states.


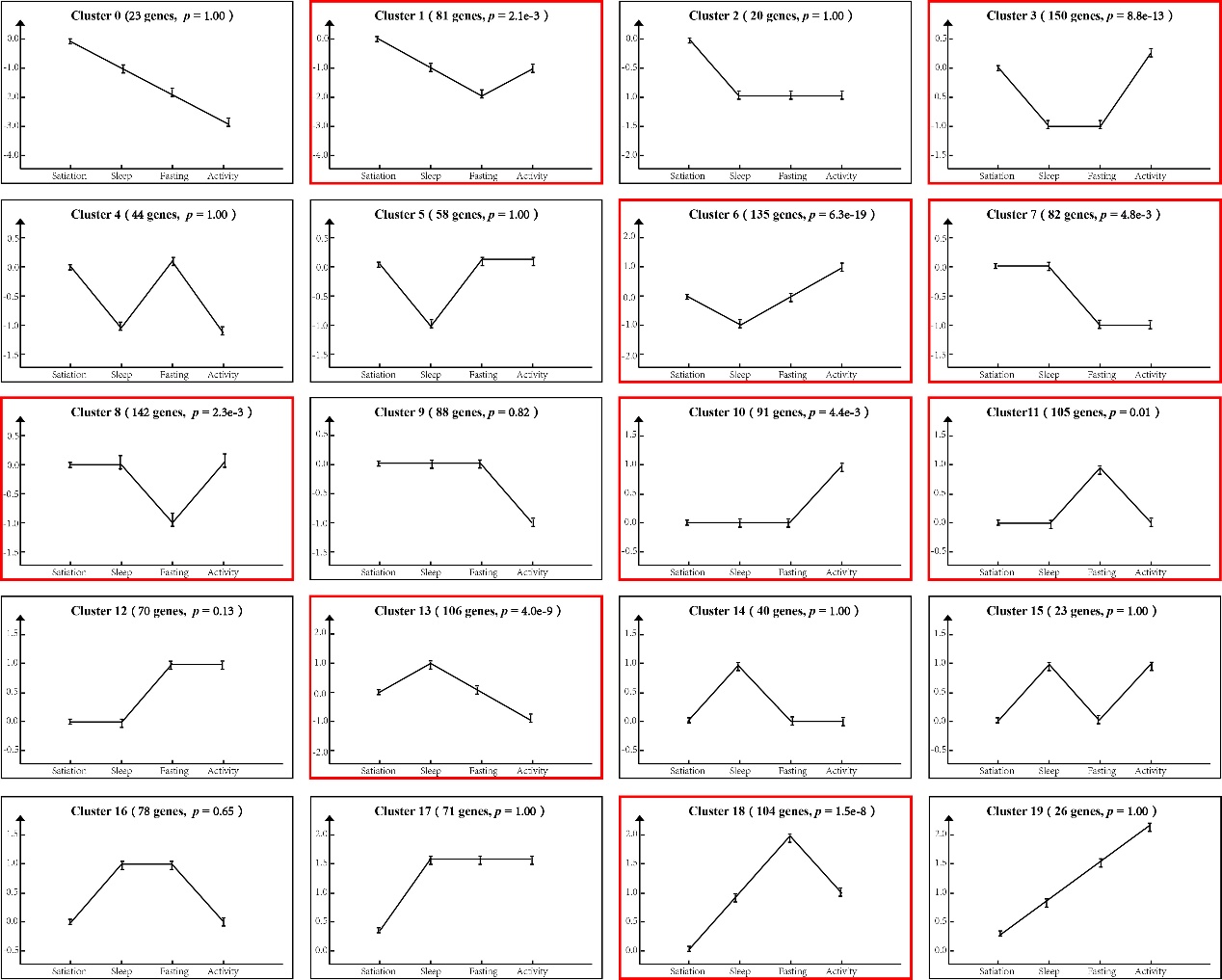


**Figure S3.** Differential gene cluster expression trend line chart.
